# Cancer Stem Cells: A Potential Breakthrough in HCC-Targeted Therapy

**DOI:** 10.3389/fphar.2020.00198

**Published:** 2020-03-06

**Authors:** Yafei Wu, Jigang Zhang, Xue Zhang, Heming Zhou, Gaolin Liu, Qin Li

**Affiliations:** Department of Clinical Pharmacy, Shanghai General Hospital, Shanghai Jiao Tong University School of Medicine, Shanghai, China

**Keywords:** cancer stem cells, hepatocellular carcinoma, signaling pathway, targeted therapy, cancer

## Abstract

Cancer stem cells (CSCs) are subpopulations of cells with stem cell characteristics that produce both cancerous and non-tumorigenic cells in tumor tissues. The literature reports that CSCs are closely related to the development of hepatocellular carcinoma (HCC) and promote the malignant features of HCC such as high invasion, drug resistance, easy recurrence, easy metastasis, and poor prognosis. This review discusses the origin, molecular, and biological features, functions, and applications of CSCs in HCC in recent years; the goal is to clarify the importance of CSCs in treatment and explore their potential value in HCC-targeted therapy.

## What Are CSCs?

**FIGURE 1:**
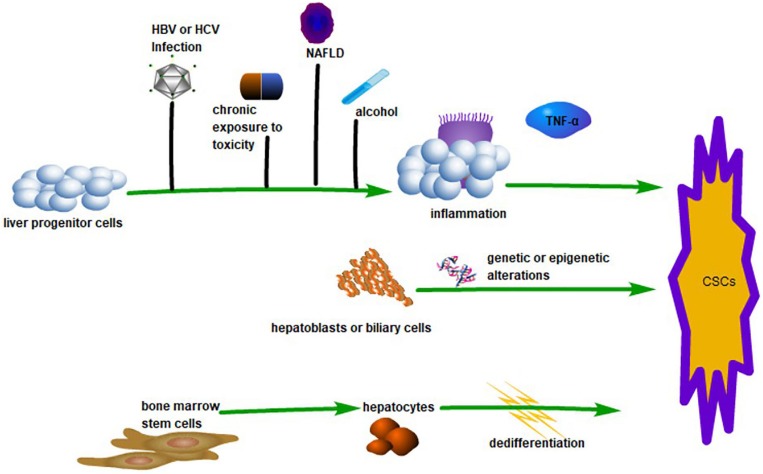
The Origin of CSCs in HCC. LPCs can transform into CSCs linked with inflammation caused by various factors such as HBV or HCV infection, alcohol, chronic exposure to toxicity, and non-alcoholic fatty liver disease (NAFLD). This process is associated with TNF-α. Hepatoblasts or biliary cells can transform into CSCs by genetic or epigenetic changes. Hepatocytes derived from bone marrow stem cells can be dedifferentiated into CSCs.

Like stem cells in normal tissue, cancer stem cells (CSCs) are small populations of cells in tumor tissue with ‘stem cell-like’ characteristics. CSCs have the capacity to self-renew and differentiate into heterogeneous tumor cells, which are responsible for the maintenance and propagation of the tumor (Batlle and Clevers, 2017). The capability of CD34^+^/CD138^–^ cells to initiate tumors in acute myeloid leukemia was the first conclusive evidence for CSC (Bonnet and Dick, 1997). Basing on this breakthrough, CSCs were subsequently found in a variety of hematopoietic cancer and solid tumors. Hepatocellular carcinoma accounts for most of the incidence of primary liver cancer, and the existence of CSCs has been demonstrated through the identification of several surface markers in HCC (Machida, 2017). Extensive research has demonstrated that CSCs provide HCC with a proliferative, invasive, and recurrent advantage. Even so, the presence of CSCs is still controversial in HCC, which is especially evident in the theory of the origin of CSCs (see Figure 1). Some studies suggest that CSCs originate from liver progenitor cells (LPCs). The inflammatory induction of LPCs into CSCs by macrophage-secreted TNF-α represents strong evidence for this theory (Li X.F. et al., 2017). Other studies suggest that CSCs are derived from the de-differentiation of mature cells and biliary cells under the influence of genetic and/or epigenetic changes (Nio et al., 2017). More interestingly, the production of CSCs by pluripotent inducers, such as Nanog, Oct4, Yamanaka factor, and Sox2, through reprogramming is also widely accepted (Yamashita and Wang, 2013). There are also some studies that claim that CSCs are derived from bone marrow stem cells (Kim et al., 2010). Faced with the controversy over the origin of CSCs, researchers tried to explore the origin of CSC using *in vitro* culture and immunodeficient tumor models. For example, sphere cells that originate from external culture and fusion cells, which originate from cancer cells and stem cells, are deemed to be CSCs (Wang R. et al., 2016). However, questions remain as to whether CSCs induced *in vitro* are consistent with CSCs in tumors *in vivo* (Magee et al., 2012). On the one hand,

in an experiment where the tumor microenvironment was absent, a gap between the environment and the evolution of CSCs in the body would be present, which may not reflect the actual conditions *in vivo*. On the other hand, immunodeficient tumor models are different from the human immune environment. Future research should be designed in more biologically appropriate environments and more suitable immunodeficient models.

## Biological Characteristics of CSCs in HCC

### Self-Renewal

Self-renewal refers to the production of one or more cellular subtypes that retain maternal features and functions after the symmetrical and asymmetrical division of stem cells. There are a few CSCs in cancer, which can regenerate cancer cells through self-renewal (Lobo et al., 2007). To treat HCC, many researchers start by regulating CSC self-renewal. Current research has made some progress in the maintenance and regulation of a variety of intentional molecules involved in the self-renewal of CSCs. These molecules affect the expression of CSCs genes, regulate signaling pathways, and thus affect self-renewal ability. In addition, studies suggest that the tumor microenvironment inhibits CSC self-renewal. When the tumor microenvironment is altered, it affects the self-renewal and proliferation of CSCs, leading to HCC. In short, self-renewal is the most fundamental characteristic of CSCs; therefore, suppressing self-renewal of CSCs may fundamentally solve the problem of tumorigenesis and expansion.

### Differentiation

Cancer stem cells produce the same tumorigenic cells through self-renewal and grow into non-tumorigenic cancer cells through differentiation, promoting tumor proliferation (Reya et al., 2001). Researchers have shown that CSCs can differentiate into different tumor cells in liver cancer through monoclonal experiments (Liu et al., 2013). CSCs multi-directional differentiation affects tumor heterogeneity (Huang Z. et al., 2015). Accumulating evidence suggests that CSC differentiation is associated with specific markers in HCC. For example, one meta-analysis suggests that there is a link between CSCs markers and less differentiated pathological types (Flores-Téllez et al., 2017). CD24^+^cells show stronger differentiation ability than CD24^–^ cells and a higher CK18 expression can be observed in CD24^+^ cells upon differentiation (Lee et al., 2011). Knockout CD44 promotes CSCs to differentiate to a normal cell-like morphology (Han S. et al., 2015). A study has revealed that Li-7 HCC cells maintain a clearly heterogeneous hierarchy and instability based on CD13^+^ CSCs differentiation (Yamada et al., 2015). Moreover, specific markers such as EpCAM (Yamashita et al., 2009) and CD133 (Ma et al., 2007) can be utilized to identify CSCs based on their differentiation stage. During CSCs differentiation, Histone deacetylatase sirtuin 1(SIRT1) expression decreases while endogenous Nanog decreases and albumin increases (Liu et al., 2016). β2 spectrin has a positive effect on CSC differentiation while inhibiting the CSCs properties (Chen Y. et al., 2018). These may provide new perspectives on anti-CSCs differentiation strategies.

### Cell Autophagy

Autophagy is the degradation and removal of endogenous proteins and damaged organelles by lysosomes. Recent studies have found a close relationship between autophagy and CSCs in HCC. In terms of gene expression, autophagy promotes CSC characteristics by inhibiting p53 expression (Liu et al., 2017b). In terms of molecular characteristics, autophagy is related to Axin2^+^ CD90^+^ CSC induction (Li J. et al., 2017). In terms of biological characteristics, CD133^+^ CSCs escape the living pressure caused by the lack of nutrition and the hypoxic environment of HCC by means of autophagy, especially low glucose concentrations (Chen H. et al., 2013). Interestingly, it has been demonstrated that autophagy provides the decomposable metabolites needed for repair, removes toxic substances, and reduces cytoplasmic acidification to contribute to the survival of CD133^+^ CSCs in response to hypoxia and nutrient starvation stress (Song et al., 2013; Nazio et al., 2019). In addition, it was found that 3-methyladenine and bafilomycin A1 significantly reduced the number of CD133^+^ CSCs and ball formation ability, which are related to autophagy (Liu et al., 2017b). Altogether, autophagy is clearly evidenced in the tumor initiating and drug resistance capabilities of CSCs (Garcia-Mayea et al., 2019). The development of autophagy inhibitors will revolutionize the appearance and maintenance of CSC stemness (Li and Zhu, 2019), and autophagy inhibitors alone or combined with existing chemotherapeutic drugs will play an important role in HCC formation and drug resistance.

## The Role of CSCs in HCC

### CSCs INITIATE HCC

In 2007, the infinite value-added accumulation of CSCs was proposed to trigger HCC. A hierarchical model was proposed in 2010 to indicate that apical CSCs are responsible for the initiation of primary cancer. Years of theory have continually optimized and confirmed that CSCs are tumorigenic after division and differentiation. The side population (SP) cells were isolated from human HCC cell lines MHCC97-H, MHCC97-L, Huh7, and HCCLM3 and transplanted into murine-induced murine HCC130, which provided sufficient evidence for the ability of CSCs to initiate tumorigenesis. In summary, the occurrence of HCC is closely related to CSCs, and the specific mechanism has not yet been elucidated. Targeting CSCs to inhibit tumorigenesis will contribute to the treatment of HCC.

### CSCs Affect the Malignant Features of HCC

#### CSCs Affect HCC Metastasis

The effects of CSCs on HCC metastasis, drug resistance, prognosis, and relapse have been confirmed in recent years. CSCs may be part of the critical drivers of HCC metastasis with inextricable elements including self-renewal and the tumor-initiating ability of CSCs (Lambert et al., 2017). The plasticity of CSCs and the promotion of EMT activity are also important causes of HCC metastasis (Wang S.S. et al., 2015). CSC markers and the EMT phenomenon are closely linked to the metastasis of HCC. ROS enhances tumor metastasis *via* migration, invasion, and angiogenesis (Lee et al., 2019) while CD13 overexpression effects metastasis by reducing ROS *via* an EMT phenomenon (Kim et al., 2012). Studies have found that Notch inhibitor PF-03084014 inhibits the self-renewal and proliferation of CSCs and further inhibits HCC metastasis, which is evidence of the potential application of gamma-secretase inhibitors in a targeted therapy for HCC (Wu C.X. et al., 2017). Sorafenib inhibits CD90^+^ CSCs and extracellular vesicle production to inhibit distant HCC metastasis (Yoshida et al., 2017). Knocking out CD44 *in vivo* and *in vitro* is beneficial in suppressing tumor metastasis. This process may be related to EMT reversal and the ERK/Snail pathway (Gao et al., 2015). The linkage between special CSC markers and the EMT phenomenon provides a potential therapeutic perspective against HCC metastasis.

#### CSCs Affect HCC Drug Resistance

It is worth mentioning that the plasticity of CSCs is also one of the things that affects HCC drug resistance. Another effect related to drug resistance is the fact that CSCs can quickly mediate toxic efflux and rapidly respond to oxidative stress and DNA damage. Furthermore, some markers and RNA associated with CSCs can be potential targets of defeating resistance to chemotherapy. For example, sorafenib resistance may be associated with Nanog^+^ CSCs (Chen C.L. et al., 2016), whereas lncRNA THOR inhibits CSCs and increases HCC sensitivity to sorafenib (Cheng et al., 2019). In terms of prognosis, studies have shown that CSC heterogeneity promotes HCC molecular and biological diversity, leading to a poor prognosis. In addition, CSCs may be used to assess prognosis, such as CSCs-associated DKK1 mRNA as a prognostic indicator for HCC.

#### CSCs Affect HCC Recurrence

In terms of recurrence, CSCs have greater resistance to chemotherapeutic drugs, stimulate invasion through EMT, and can survive and reoccur after treatment (Cheng Z. et al., 2016). Stable overexpression of miR-216a/217 induced EMT increased the CSC population of HCC. Circulating miR-1246 has been shown to be a predictor of survival and tumor recurrence in HCC patients after liver transplantation (Xia et al., 2013). Interactions between CSCs and angiogenesis should be attributed to the recurrence and angiogenic treatment resistance of patients with HCC. Chemoradiotherapy may induce non-CSCs to differentiate into CSCs, causing tumor recurrence (Chen X. et al., 2017). CSC enrichment and proliferation induced by stress also points to a mechanism for recurrence in HCC (Huo et al., 2019). In addition, β-catenin signaling is associated with tumor malignant differentiation and is involved in tumor recurrence. Changes in IL-6 concentration in the tumor microenvironment promote tumor invasion and metastasis and participate in recurrence. Studies have confirmed that some markers are closely related to HCC recurrence. For example, CD13^+^ CSCs form cell clusters along the fibrous envelope, which is closely related to the recurrence of HCC after TAE (Haraguchi et al., 2010). The recurrence rate of patients with a high CD133 expression is higher than that of a low CD133 expression (Song et al., 2008). The process of CD133^+^ CSCs promoting the recurrence of HCC is closely associated with VEGF (Liu et al., 2017a). Other studies have shown that CD44 expressions in non-tumor tissues may predict HCC recurrence (Tovuu et al., 2013). Presently, acyclic retinoid (600 mg/d) targets MYCN^+^ CSCs and successfully reduces the 2-year recurrence rate after liver cancer treatment (Qin et al., 2018). In short, CSCs play an important role in HCC progression. This suggests that more effort should be put into clarifying the molecular mechanisms and developing targeted drugs for the treatment of HCC.

#### CSCs and Epithelial-Mesenchymal Transition

EMT refers to the process in which epithelial cells lose cell polarity and intercellular adhesion and obtain migration and invasiveness capabilities as mesenchymal stem cells. The EMT-related genes and the K19^+^ CSC gene are jointly expressed in HCC (Kawai et al., 2015). Exogenous overexpression of Twist2 is associated with EMT enhancement of CSC-related gene expression such as BC-1, Sox2, and Nanog, which improves CD24^+^ CSCs self-renewal ability (Liu A.Y. et al., 2014). This is also one of the examples of joint expression. Moreover, EMT-related factors promote the expression of CSCs marker (Park et al., 2016) and the development of CSCs. In addition, EMT activation confers greater invasiveness and resistance to CSCs and promotes tumor recurrence (Ikemoto et al., 2017).

#### CSCs and Angiogenesis

Cancer stem cells participate in angiogenesis; for example, CSCs initiate tumor angiogenesis *via* the lateral differentiation of EMT or angiogenic factors and CD133^+^ CSCs with abnormal IL-8, NTS, and CXCL1 expression induces angiogenesis (Yang et al., 2010). Additionally, CD90^+^ CSCs release exosomes containing lncRNA H19 and regulate the angiogenic phenotype (Conigliaro et al., 2015).

Vasculogenic mimicry (VM) refers to an invasive tumor cell that mimics the embryonic angiogenic vascular network. CSCs may be involved in the formation of VM, affecting the reverse transformation between VM and endothelium dependent blood vessels (Fan et al., 2013). In different differentiated states of HCC, CSC gene expression has different effects on VM formation. Slug (SNAI2) (Sun et al., 2013), lncRNA n339260 (Zhao et al., 2018), Twist, c-Myc, and Sox2 are key factors for CSCs to promote VM.

In conclusion, CSCs play a role in angiogenesis and vascular mimicry, which are related to malignant features such as HCC metastasis and recurrence. Therefore, the development of targeted agents for CSCs to inhibit tumor blood supply, and the combination of the original radiotherapy and chemotherapy drugs, can improve the effectiveness of HCC treatment.

## CSC Markers and Heterogeneity in HCC

Cancer stem cells, with a strong ability to self-renew and strong tumorigenicity, are isolated from tumor cells, cultured, and then implanted into mouse models to produce new tumor masses. Based on this technique, both accuracy and efficiency are of primary concern. In order to improve separation quality, CSC specific markers are used for fluoresce-activated cell sorting (FACS) as this method can obtain a purer CSC population. The search for specific CSC markers has therefore become a new target for researchers. In recent years, researchers have found a variety of markers that are expressed by CSCs (see Table 1). Specifically, CSC markers can be summarized by four functions: self-renewal, differentiation, proliferation, and tumorigenic ability (Wang et al., 2018). For example, EpCAM promotes CSC self-renewal and differentiation (Yamashita et al., 2009), keratin 19 (K19) enhances the proliferation of CSCs (Kawai et al., 2015), and overexpression of the calcium channel α2δ1 increases the tumorigenic capacity of CSCs (Zhao et al., 2013).

**TABLE 1:** Different markers associated with CSCs in HCC.

**Markers**	**Functions in CSCs**	**Signaling pathway**	**References**
EpCAM	Drug resistance, tumorigenesis, invasion, self-renewal	Wnt/β-catenin	Terris et al., 2010
Calcium channel α2δ1	Calcium influx	ERK	Zhao et al., 2013
CD133	Drug resistance, tumorigenesis, self-renewal, proliferation, angiogenesis	Akt/PKB, Neurotensin/IL-8/CXCL1	Ma et al., 2008; Tang et al., 2012
CD90	Drug resistance, tumorigenesis, self-renewal	PI3K/Akt1, TGF-β	Yamashita et al., 2013; Zhang et al., 2015
CD24	Drug resistance, tumorigenesis	STAT3-mediated NANOG regulation	Lee et al., 2011
K19	Proliferation, EMT, drug resistance, invasion	Smad/TGF-β	Kawai et al., 2015
CD44	Regulation of redox status through xCT, self-renewal, drug resistance, maintenance, tumorigenesis	ROS-induced stress Notch3	Ishimoto et al., 2011; Asai et al., 2019

In addition, phenotypic heterogeneity of CSCs refers to the fact that they express a variety of different stem cell markers, which can be used to identify and isolate CSCs as well as represent different clinical and prognostic significances. In addition, CSCs showed significant heterogeneity in self-renewal and differentiation potential (Zheng et al., 2018). Nevertheless, the phenotypic heterogeneity of CSCs not only bring some difficulties to the accurate separation and identification of CSCs, but the existence of multiple phenotypes is also not conducive to the efficiency and universality of modeling. Studies have found that the heterogeneity of CSCs (Tang, 2012; Flores-Téllez et al., 2017) is not limited to biological phenotypes. There is also some heterogeneity in transcriptomics, such as karyotype evolution and gene expression profiles (Colombo et al., 2011). However, this is limited to the single-cell level, and the transcriptome heterogeneity of CSC groups is significantly reduced. This is also a controversial topic in CSC transcriptional heterogeneity.

Intra-tumor heterogeneity is closely related to genetic and functional diversities and is highly complex in HCC (Prasetyanti and Medema, 2017). Until now, more than a dozen markers, such as CD130, CD24. CD90, CD13, EpCAM, and K19 have been identified in tumor cell populations, demonstrating the phenotypical heterogeneity of tumor cells. The expression of different markers is related to prognosis, metastasis, recurrence, and drug resistance of HCC, which poses certain difficulties in the treatment of HCC. In terms of prognosis, researchers applied 18F-fluorodeoxyglucose positron emission tomography (18F-FDG-PET) to provide a more accurate prognostic prediction based on the association between K19 and poor prognosis of HCC (Kornberg and Friess, 2019). In addition, the application of CD133 in prognostic prediction should also be noted. CD133 in the cytoplasm of CSCs indicates poor prognosis, while CD133 in the nucleus indicates the opposite. This finding provides an important theoretical basis for the future prediction of prognosis, based on the sub-localization of CSC markers (Chen Y.L. et al., 2017). There is no doubt that many markers, especially CD13, CD44, and K19, affect HCC drug resistance. Further research has found various mechanisms by which markers affect resistance. For example, CD13 blocks HCC apoptosis under the influence of genotoxic chemotherapeutic fluorouracil (Haraguchi et al., 2010). The effect of CD44 on antioxidant capacity is related to glutathione peroxidase 1 (GPX1) and thioredoxin (Asai et al., 2019). Furthermore, K19 is involved in endothelial-mesenchymal transition (EMT) and TGF-β signal transduction to regulate drug resistance. A better understanding of the mechanism of marker-related drug resistance will greatly assist the future development of targeted drugs.

Cancer stem cell markers are also important in other aspects of HCC, such as metastasis and recurrence. Future studies on targeted CSC therapy can focus more on K19, CD90, CD44, Toll-like receptors 4 (TLR4), SRY-related HMG-box gene 12 (SOX12), and aldehyde dehydrogenase (ALDH) to inhibit tumor metastasis. At the same time, more attention should be paid to CD133, K19, CD13, and TLR4 to cope with HCC recurrence. The accurate isolation and identification of CSCs is of great benefit to the targeted CSC therapy of HCC. Using CSC markers is currently a mainstream practice. Furthermore, CSC markers also open the way for exploring other potential biological functions and characteristics of CSCs.

## Factors Regulating CSC Function in HCC

The acquisition and maintenance of CSC characteristics are regulated by many factors. Although the detailed mechanisms are not clear, the regulatory roles of gene expression, tumor microenvironment, and multiple signaling pathways is beyond a doubt.

### Gene Expression and Epigenetics

A single-cell gene analysis found that the characteristics of CSCs are related to the expression of many genes. For example, the maintenance of tumorigenicity of CSCs is related to the BC047440 gene, and NF-κB and HNF4 may be key regulators (You et al., 2014). Other examples show that the self-renewal ability of CSCs is related to genes C8orf4 (Zhu et al., 2015), BMI1, p53, Numb and p53, and Numb may form an expression network where one interacts with the other (Siddique et al., 2015). Alterations in stemness genes, related transcription factors, and proteins affect CSC characteristics. All of these provide new insights into the development of potential drug targets. Future research efforts should focus on the development of characteristic gene regulatory molecules and more in-depth mechanisms to improve the effectiveness and safety of targeted therapies.

Epigenetic changes are inextricably linked to CSC phenotypes, HCC biological behaviors, and patient clinical outcomes. There have been certain breakthrough in the research on the regulation of CSCs in terms of DNA modification, histone modification, non-coding RNA regulation, and chromatin remodeling. For example, DNA methylation regulates the development and application of CSC tumorigenicity-inspired methylation inhibitors DNMT1 and DNMT3. The transcriptional repressor SALL4 is modified by deacetylation to promote the overactivation of CSCs (Zeng et al., 2014). The effect of BMI-1 on the properties of CSCs may be accomplished by means of chromatin changes. In recent years, non-coding RNA has become a hot topic in research and the results are remarkable (see Tables 2, 3). In short, intervention and regulation of epigenetic regulatory factors may have a positive effect on the development of probes to accurately identify CSCs and the development of novel targeted drugs. The commonalities and differences between traditional gene expression and epigenetic changes in CSCs have not been perfectly explained. These two points require further efforts from researchers.

**TABLE 2:** Different microRNAs associated with CSCs in HCC.

**MicroRNA**	**Impact on CSCs**	**References**
miR-122	Inhibits the growth of CD133^+^ CSCs and inhibits tumor stemness	Song et al., 2015
miR-150	Inhibits the subgroup of CD133^+^ CSCs	Zhang et al., 2012
miR-152	Inhibits CD133^+^ CSCs cloning and growth	Huang H. et al., 2015
miR-613	Inhibits CD24^+^ or OV6^+^ self-renewal and amplification	Li et al., 2019
miR-200	Inhibits tumor stemness	Wang J. et al., 2015
miR-let-7	Inhibits self-renewal and gene expression	Han H. et al., 2015
miR-155	Influences phenotypic expression	Han et al., 2012
miR-429	Promotes self-renewal, tumorigenicity and chemical of EpCAM^+^ CSCs	Li et al., 2015
miR-1246	Promotes tumor stemness	Chai et al., 2017
miR-449a	Promotes tumor stemness	Zhang Q. et al., 2017
miR-25	Promotes proliferation	Feng et al., 2016
miR-21	Promotes invasion and migration	Jiang et al., 2016
miR-16	Inhibits drug resistance	Qiu et al., 2019

**TABLE 3:** Different lncRNAs associated with CSCs in HCC.

**lncRNA**	**Impact on CSC**	**References**
lnc Sox4	Promotes self-renewal	Chen Z.Z. et al., 2016
lnc TCF7	Promotes self-renewal	Wang Y. et al., 2015
lnc β-Catm	Promotes self-renewal	Zhu et al., 2016a
lnc BRM	Promotes self-renewal and tumorigenicity	Zhu et al., 2016b
lnc THOR	Low expression promotes self-renewal and expansion, reduces drug resistance	Cheng et al., 2019
lnc CUDR	Promotes self-renewal and amplification	Pu et al., 2015
lnc ARSR	Promotes amplification	Yang et al., 2019
lnc HULC and lnc MALAT1	Promotes proliferation in a coordinated way	Wu et al., 2016
lnc HOTAIR	Promotes proliferation and non-CSC transformation to CSC	Wu L. et al., 2017
lnc CAMTA1	Promotes tumor stemness	Ding et al., 2016
lncHAND2-AS1	Promotes self-renewal	Wang et al., 2019

### Tumor Microenvironment

In HCC, the tumor microenvironment regulation of CSCs is associated with multiple signaling pathways, cancer-associated fibroblasts (CAFs), and tumor- associated macrophages (TAMs). On the one hand, more than 50% of CAFs are shown to be CD90^+^ CD44^+^ (Yamashita and Wang, 2013) and promote CSCs through HGF-mediated cMet/FRA1/HEY1 signaling (Sun et al., 2019). On the other hand, TAMs induce IL-6 to activate the STAT3 pathway and promotes the growth of CSCs. M2 TAMs secrete TNF-α, activate the Wnt/β-catenin pathway in SMMC-7721 hepatoma cells, and induce the appearance of EMT and CSCs (Chen et al., 2019). Even more surprisingly, TAMs promote CSC-like properties *via* TGF-β1-induced EMT and they may contribute to the prognosis of HCC (Fan et al., 2014). This suggests that the combination of CSCs and TAM can be a new target for HCC treatment. The hypoxic state of the tumor microenvironment is closely related to the activity of CSCs, and HIF-1α may be an inducer (Muramatsu et al., 2013). In addition, abnormal expression of CSCs under hypoxia promotes malignant activation and the tumorigenesis of CSCs. This is particularly evident in the abnormal expression of angiogenesis-related genes, cell signaling, structure, metabolism, growth, and other related genes (Choi et al., 2017).

### Multiple Signal Pathways

#### TGF-β

TGF-β signaling is involved in controlling the occurrence, differentiation, and maintenance of CSCs. As early as 2013, researchers found that in CD133^+^ CSCs, TLR4 targeting Nanog inhibits the tumorigenicity and drug resistance of CSCs and is associated with TGF-β abnormalities. However, simultaneous silencing of YAP1 and IGF2BP3 restored TGF-β signaling (Chen C.L. et al., 2013). In 2016, a study revealed that TGF-β affects the tumor microenvironment of CSCs, especially CSCs under the stimulation of reactive oxygen species (Carnero and Lleonart, 2016). A year later, the effects of TGF-β on EMT and plasticity of CSCs were clearer. Both TGF-β and TNF-α can promote the transformation of non-CSCs into CSCs, as well as promote the self-renewal and tumorigenic effects of CSCs phenotypes in HCC cell lines (Malfettone et al., 2017). Throughout the past 2 years, more studies have revealed the abnormal expression of genes in this signaling pathway, such as the gene c-Myc and Sox2 expression activation signaling pathway and mitotic cell cycle regulation. Undoubtedly, TGF-β is currently the most promising target, prompting researchers to explore the detailed mechanisms in this signaling pathway.

#### Akt

The Akt signaling pathway is involved in the regulation of CSC homeostasis and drug resistance. There are several key molecules in the Akt pathway, such as EGFR, PI3K, GSK-3β, β-catenin, etc. In the EGFR/Akt signaling pathway, CD133 affects EGFR internalization, makes EGFR unstable, inhibits EGFR/Akt signaling, and affects drug resistance and tumorigenicity (Jang et al., 2017). While PI3K/Akt signaling mediates the formation of CSCs, the initiation of HCC occurs and mTOR is the downstream molecule (Gedaly et al., 2013). In addition, the study also found that HBV X protein promotes alpha-fetoprotein (AFP) expression and relies on PI3K/Akt signaling to promote CSC proliferation (Zhu et al., 2017). At the same time, the inhibitory effect of 2-morpholino-8-phenyl-4H-chromen-4-one (LY294002) and 5-fluorouracil (5-FU) on CD90 ^+^ CSCs also depends on PI3K/Akt signal (Peng et al., 2016). At present, the study found that Akt/GSK3 β/β-catenin promotes the proliferation and invasion of CSCs (Xu et al., 2017). Unfortunately, inhibition of Akt activity reduces CSCs self-renewal accompanied by multiple adverse events such as liver damage, inflammation, hyperglycemia, and hyperinsulinemia (Wang et al., 2017). Targeting Akt against CSCs (Liu L. et al., 2014), in addition to elucidating a more detailed mechanism of action to improve the effectiveness of treatment, should also be considered in reducing the incidence of adverse drug reactions.

#### Notch

Notch regulates the biological properties of CSCs, of which Notch1 and Notch3 are particularly prominent. On the one hand, Notch1 regulates the expression levels of p53, p21, and p27 by HES1 and cyclinE, and affects the self-renewal and expansion of CD90^+^ CD133^+^ CSCs (Zender et al., 2013). Mechanistically, the Notch1 intracellular domain of NICD1 activation is dependent on Wnt/β*-*catenin, and there is a non-proteasome-mediated regulatory loop between both (Wang R. et al., 2015). Even the anti-tumor and metastatic ability of Notch inhibitor PF 03084014 was associated with Notch1 inhibition, signal pathway inactivation, and decreased EMT (Wu C.X. et al., 2017). On the other hand, Notch3 as a positive regulator of CSCs has a negative correlation with the expression level of β-catenin and synergizes with it to regulate the characteristics of CSCs in HCC. Although we still wonder whether the interaction mechanism between Notch3 and β-catenin signaling is the same as that of Notch1, we can determine the mechanism of action that clarifies the coordination of multiple signaling pathways to better target CSCs in the treatment of HCC.

#### STAT3

In MHCC97-L cells, STAT3/Nanog pathway activation promotes self-renewal and extensive proliferation of CSCs, which improves drug resistance and tumorigenicity (Yin et al., 2015). In other subtypes, the effects of various substances such as human growth hormones (Chen Y.J. et al., 2017), lnc ARSR (Yang et al., 2019), and miR-500a-3p (Jiang et al., 2017) on CSCs are also dependent on the STAT3 signaling pathway, but the mechanisms vary. The abnormal expression of human growth hormones (hGH) is dependent on hGH-STAT3-CLAUDIN-1 and miR-500a-3p targets multiple negative regulators such as SOCS2, SOCS4, and PTPN11 in the JAK/STAT3 signaling pathway. In conclusion, STAT3 signaling plays a key role in CSC self-renewal, tumorigenicity, and resistance. Deepening the understanding of the STAT3 molecular mechanism and finding suitable targeting molecules will open new avenues for targeted therapy.

#### Wnt

Wnt signaling maintains CSCs’ self-renewal capacity, inhibits CSC differentiation, and is associated with CSC induced resistance. β-Catenin is one of the molecules downstream of the Wnt signaling pathway. Mechanistically, the Wnt ligand binds to the Frizzled protein, which triggers cytoplasmic β*-*catenin accumulation; β–catenin-incorporated TCF/LEF molecules induce transcriptional activation of Wnt target genes (Chen D. et al., 2018). Furthermore, Shp2 (Xiang et al., 2017) and ethanol molecules may be promoters of Wnt/β-catenin signaling, and MYCN may be a signal regulator (Qin et al., 2018). STAT3 may be another downstream molecule of the Wnt signal, and chitosan is involved in activating non-canonical Wnt/STAT3 signaling, which induces CD44^+^ CSCs production (Chang et al., 2017). The development of several downstream molecules targeting the Wnt signaling pathway (Pez et al., 2013), especially β-catenin and STAT3 inhibitors, will bring hope to targeted therapies. However, this also inevitably affects other signal pathways. Therefore, the more detailed regulatory mechanisms should also be clarified.

#### Other Signal Pathways

In recent years, research has also progressed in other CSC signaling pathways. Researchers have a good understanding that MEK (Cheng J. et al., 2016) and JNK (Tong et al., 2015) regulate CSC self-renewal; Hedgehog (Jeng et al., 2013), NF-κB/snail (Nikolaou et al., 2015), IL-33/p38 (Xie et al., 2019), and ERK1/2 (Mahati et al., 2017) are related to CSC features; and ERK is also involved in the migration and invasion of CSCs (Sun et al., 2017), providing another explanation for the transfer mechanism of HCC. Although these signal pathway mechanisms vary widely, they have made outstanding contributions to the regulation of CSCs. Combining multiple signaling pathways to find hub molecules in signaling pathway networks may provide a new perspective for targeted therapies (Clara et al., 2019).

**FIGURE 2:**
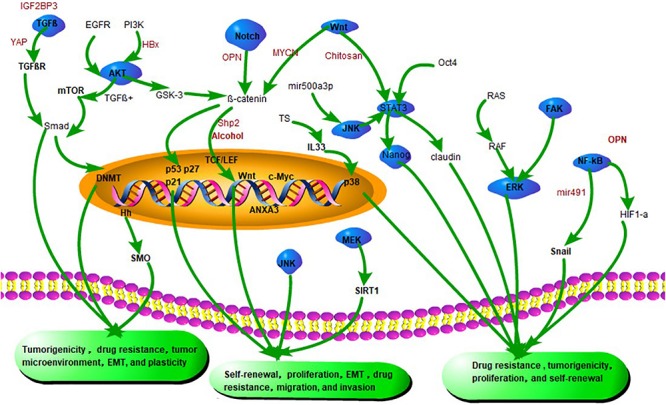
Emerging signaling pathways in CSCs. Silencing of both YAP1 and IGF2BP3 restores TGF-β signaling and eliminates drug resistance of CSCs. HBV X protein (HBx) promotes the initiation of CSC by promoting the expression of α-fetoprotein through the PI3K/AKT signaling pathway. Shp2 promotes CSC expansion and prognosis by activating β-catenin signaling. β-catenin interacts with TCF/LEF factors and induces transcriptional activity of Wnt signaling genes such as cyclin D1, c-Myc, and surviving. Chitosan promotes stem cell properties associated with Wnt-STAT3 signaling. miR-491 reduces the CSC-like properties of HCC via the NF-κB signaling pathway. Inhibition of miR-21 attenuates osteopontin (OPN) expression by blocking Notch and its downstream target transcription factor RUNX2/HES1. OPN also promotes CSC-like phenotypes via the integrin αvβ3–NF-κB–HIF-1α pathway.

In summary, CSC characteristics are regulated by multiple signaling pathways (see Figure 2). Elucidating the signaling pathway mechanisms and developing key molecular targeted agents will bring hope for curing HCC.

## Targeting CSCs Therapy Strategies and Potential Value

**FIGURE 3:**
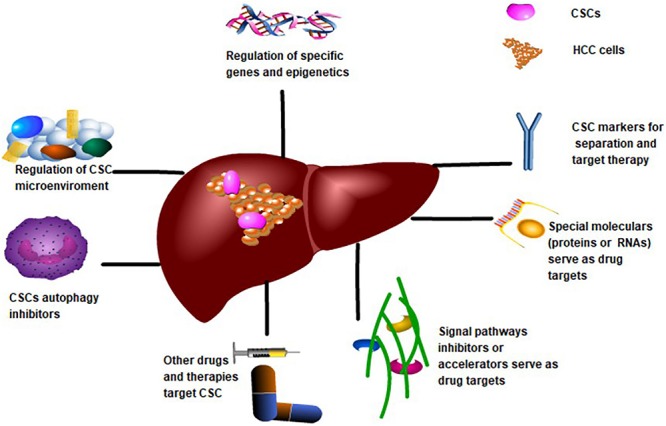
Targeting CSCs Therapy Strategies for CSCs to treat HCC. CSCs markers and specific proteins or RNAs may be potential targets for drug development. Regulation of CSCs-related gene expression or epigenetics is also a potential target. The application of other compounds and biotherapy is beneficial for targeting CSCs. Targeting CSCs-related signaling pathways, CSCs microenvironment and inhibition of CSCs autophagy are also important directions of targeted therapy.

Presently, research views CSCs from a variety of perspectives (see Figure 3). CSC markers like CD40 and CD90 are the target standards for separation and drug targeting (Yoshida et al., 2017). Additionally, targeting the tumor microenvironment on which CSCs depend, such as the blood supply and metabolism, inhibits the growth and differentiation of CSCs. Epigenetic regulation of CSC gene expression by histone modification and methylation is thought to be promising. In contrast, the key signaling pathways that regulate CSCs, such as Wnt/β*-*catenin, Notch, and STAT3 affect the characteristics of CSCs. Furthermore, the development of CSC vaccines for immunotherapy, the promotion of CSCs after differentiation and chemotherapy and radiotherapy, stimulation of quiescent CSCs into the cell cycle, and the micro/nano-targeting of CSCs are potential therapeutic strategies for HCC (Locatelli et al., 2019). Contemporary mainstream targeted therapies are based on the molecular or biological properties of CSCs.

Moreover, endocrine hormones such as thyroid hormones (Catalano et al., 2016) and dopamine affect the characteristics of CSCs (Garcia-Mayea et al., 2019). The thyroid hormone increases the proportion of CD90^+^ CSCs, promoting CSCs self-renewal and tumorigenicity. TRα interacted with p65 induces BMI1 expression by binding to the promoter region of the BMI1 gene, revealing that TH signal plays an important role in regulating CSC self-renewal, by activating the NF-κB signaling pathway (Wang T. et al., 2016). Dopamine enhances the expression of EMT markers (*N*-cadherin, Vimentin) and sternness markers (Nanog, SOX2, and OCT3/4) in HepG2 cells. Dopamine promotes EMT and stemness of HCC by inducing the expression of SULT1A3/4 (Zou et al., 2017) and may provide a new strategy for the clinical targeted therapy of HCC.

Bis(2-ethylhexyl) phthalate is a carcinogen of HCC while curcumin may be a potential antidote to phthalate-induced HCC progression. Curcumin may inhibit acyl hydrocarbon receptor/ERK/SK1/S1P3 signaling, inhibit phthalate-induced cell migration, invasion, and EMT, reduce the proportion of CSCs in hepatoma cell lines *in vitro*, and inhibit the growth and metastasis of HCC (Tsai et al., 2015). The antipsychotic drug pimozide inhibits stemness and tumorigenesis of SP cells and CD133^+^ cells, inhibiting the proliferation and migration of HCC. Pimozide blocks EMT and affects the differentiation of CSCs by inhibiting Wnt/β -catenin signaling (Tsai et al., 2015). Cantharidin has certain effects on proliferation, autophagy, cell cycle arrest, and apoptosis induction of CSCs in the HepG2 cell line. This process is associated with the phosphorylation of histones H2AX, Myt1, cyclin A2, cyclin B1, p53 and Tyr15. Nicloxamide inhibits stress induced by local treatment and stimulates the enrichment, proliferation, and self-renewal of CSCs (Tsai et al., 2015). Arsenic trioxide (ATO) significantly reduced the characteristics of CSCs. The expression of the minichromosome maintenance protein (MCM) 7, a potential target of ATO, is raised in HCC, which is significantly correlated with tumor size, cell differentiation, portal vein embolism, and poor patient survival. ATO inhibits MCM7 transcription and CSC metastasis by targeting the serum response factor (SRF)/MCM7 complex (Tsai et al., 2015). Even non-mainstream treatments, such as bioviral oncolytic adenovirus GD55, target CSCs to treat HCC. Biotherapy uses the genetically modified oncolytic adenovirus to selectively enter and spread into HCC, generating cytotoxicity and tumor inhibition. Oncolytic adenovirus GD55 has a strong killing effect on CSCs, and the novel oncolytic adenovirus, carrying the tumor suppressor gene TSLC1, inhibits the Wnt signaling pathway and inhibits the growth and metastasis of CSCs *in vivo* (Zhang J. et al., 2017).

In conclusion, based on the important role of CSCs in the development of HCC, targeting CSC markers, RNAs, and signaling pathways are potential targets for targeted HCC therapy. The potential value of CSCs for the treatment of HCC can be explored through the molecular and biological characteristics of CSCs. In addition, some compounds, hormones, and biological agents can also explore the potential value of CSC targeted therapy for HCC.

## The Way Ahead and Current Challenge

To a certain extent, CSCs reveal the intrinsic reasons for the low 5-year survival rate of HCC. The scarcity of CSCs requires greater accuracy in the separation of CSCs. Currently, researchers mainly use CSC markers to separate and identify CSCs. Although the reliability of this approach remains controversial, there is no doubt that the use of surface markers to isolate and identify CSCs has broad prospects. In addition, the production, maintenance, and function of CSCs are related to many specific proteins and RNAs. Understanding the regulation of these proteins or RNAs will help in the development of new therapeutic drugs for CSC targeting.

Cancer stem cells not only initiate HCC, but also affect HCC metastasis, invasion, recurrence, drug resistance, and prognosis. CSCs are also involved in maintaining and promoting the malignant properties of HCC such as plasticity, heterogeneity, EMT, angiogenesis, and vascular mimicry. The biological characteristics of CSCs are closely related to specific gene expressions and multiple signaling pathways. This means that targeted drugs based on the molecular and biological properties of CSCs, even targeting CSCs in combination with standard therapies, provide opportunities for the complete eradication of HCC (Wang et al., 2018). Future research efforts should (1) improve the accuracy of isolation and identification of CSCs in individual HCC patients, (2) elaborate the molecular mechanisms involved in the regulation of CSCs at various tumor stages, (3) develop targeted drugs for the molecular and biological properties of CSCs, and (4) improve the effectiveness of drug therapy. In conclusion, targeting CSCs and their biological properties brings hope of a cure for HCC.

## Author Contributions

YW, JZ, GL, and QL drafted the manuscript and edited the manuscript. YW and JZ participated in preparing the figures. XZ and HZ contributed to the data collection and data analysis. All authors read and approved the final manuscript.

## Conflict of Interest

The authors declare that the research was conducted in the absence of any commercial or financial relationships that could be construed as a potential conflict of interest.
